# Pazopanib in rare histologies of metastatic soft tissue sarcoma

**DOI:** 10.3332/ecancer.2021.1281

**Published:** 2021-09-02

**Authors:** Babita Kataria, Aparna Sharma, Bivas Biswas, Sameer Bakhshi, Deepam Pushpam

**Affiliations:** 1 Department of Medical Oncology, National Cancer Institute, Badsa, Jhajjar, Haryana, 124105, India; 2 Department of Medical Oncology, All India Institute of Medical Sciences, Ansari Nagar East, New Delhi, 110029, India; 3 Department of Medical Oncology, Tata Medical Center, DH Block(Newtown),Action area I, Kolkata, West Bengal, 700160, India; † Equally contributed to this work

**Keywords:** rare STS subtypes, soft tissue sarcoma, pazopanib, metastatic STS

## Abstract

**Background:**

Uncommon histopathological subtypes account for less than 5% cases of soft tissue sarcoma (STS) and unclassified STSs comprise another 16%, these are often chemotherapy-resistant, with a dismal outcome in unresectable/metastatic disease. Prospective studies on the use of pazopanib in this cohort of patients are lacking in the literature. Here, we describe the safety and efficacy of pazopanib in rare histologies of advanced STS.

**Materials and methods:**

We conducted a retrospective study at two tertiary cancer centres in India, evaluating 33 cases of rare subtypes of STS, who received pazopanib as per institutional protocol between January 2013 and December 2019. Patients who received pazopanib for unresectable/metastatic disease were enrolled in this study for clinicopathologic features, treatment outcome and evaluation of prognostic factors.

**Results:**

Out of 33 patients, there were seven cases of undifferentiated pleomorphic sarcoma, four cases each of myxofibrosarcoma, epithelioid sarcoma and malignant peripheral nerve sheath tumour, three cases each of haemangiopericytoma and spindle cell sarcoma, two cases of haemangioendothelioma and a case each of clear cell sarcoma, retroperitoneal sarcoma, angiosarcoma and pleomorphic rhabdomyosarcoma-adult type. The objective response rate was 27%. Most of the patients (67%) received pazopanib in second or subsequent lines of therapy. The majority (70%) were started at a lower dose of 400/600 mg and only 43% of these (10/23) could be escalated to a full dose of 800 mg based on tolerance. On univariate analysis, pazopanib’s starting dose didn’t predict progression-free survival (PFS)/overall survival (OS)/response rate. At a median duration of follow-up of 18.8 months (range 1.9–150.4 months), the median PFS and median OS were 10.3 months (95% confidence interval (CI): 5.9–14.8) and 17.8 months (95% CI: 10.7–29.3), respectively. 27% of the patients experienced grade ¾ toxicities, 12% required dose modification of pazopanib and 21% needed permanent discontinuation due to toxicity.

**Conclusion:**

Our study shows that pazopanib is active in rare subtypes of STS.

## Introduction

Soft tissue sarcoma (STS) is a group of rare, yet histologically diverse groups of malignancy [1]. STS accounts for less than 1% of adult solid malignancies [2]. There are more than 70 histopathological subtypes of STS known [3]. Their rarity and histological diversity often pose a problem of inaccurate diagnosis and management [2]. Uncommon subtypes of STS include alveolar soft part sarcoma (ASPS), angiosarcoma, haemangiopericytoma/solitary fibrous tumour (SFT), haemangioendothelioma, myxofibrosarcoma (MFS), epithelioid sarcoma and clear cell sarcoma, among others [3]. Unclassified STSs include undifferentiated spindle cell sarcoma, undifferentiated pleomorphic sarcoma (UPS), undifferentiated round cell sarcoma, undifferentiated epithelioid sarcoma and undifferentiated sarcoma not otherwise specified [4]. Together, the rare subtypes account for less than 5% of STS and undifferentiated sarcomas comprise another 16% [5]. The ‘one size fits all’ theory doesn’t apply to these uncommon and unclassified histologies, as they are often resistant to conventional chemotherapy and have a relatively poor prognosis [3].

For patients with recurrent, locally advanced unresectable, or metastatic disease, therapeutic options are limited and the outcome is usually dismal in chemotherapy-resistant subtypes [3], raising the need for an alternative approach to their management.

Lately, newer advances have helped us understand the underlying mechanisms, pathogenesis and diverse biology of different STS and shifted the paradigm towards histology-driven treatment. Targeted agents and anti-angiogenic agents’ role is increasing in the management of chemotherapy-resistant histologies of STS. Angiogenesis is a final common pathway in many malignancies including many uncommon subtypes of STS. Anti-angiogenic drugs like pazopanib and sunitinib have shown activity in chemo-resistant subtypes like ASPS and SFT [6].

Anthracycline-based chemotherapy as a single agent is the standard of care as a first-line treatment of unresectable/metastatic disease [7]. There are only a few case reports and case series on the use of pazopanib in epithelioid sarcoma with conflicting data [8–10], some showing activity, and others refuting it completely. Some data are showing the activity of pazopanib in clear cell sarcoma [11].

In the current study, we have shared our experience of pazopanib in rare subtypes of STS in terms of treatment response, survival outcome and toxicities.

## Materials and methods

This is a retrospective study conducted at two tertiary cancer centres of India (viz. All India Institute of Medical Sciences, New Delhi and Tata Medical Centre, Kolkata). All patients aged ≥18 years with histopathologically proven recurrent and/or metastatic STS except synovial sarcoma and leiomyosarcoma (two most common histologies of STS based on our previous publication) [12], registered and treated in the Department of Medical Oncology at both the centres between January 2013 and December 2019 were screened. Patients who received pazopanib at any time point (either at the time of progression (previously treated with surgery/radiation therapy/chemotherapy and recurred /progressed) or upfront (de-novo unresectable/metastatic disease and unfit for chemotherapy)) were included in this study. Diagnosis of STS was made on the histopathological study of true-cut biopsy specimen with appropriate immunohistochemistry (IHC). An appropriate molecular study was performed whenever available (based on the logistics and affordability of the patient). Few cases that could not be classified based on IHC and/or molecular subtyping were labelled as spindle cell sarcoma. The Institute Ethics Committee (IEC) of both centres approved the study. Patients with incomplete medical records were excluded.

We screened case records of all patients fulfilling the inclusion criteria. Data for baseline demographic parameters, previous lines of therapy, the dose of pazopanib, toxicity were obtained and recorded. Pazopanib was administered in different doses ranging from 400 to 800 mg, once a day. The starting dose of pazopanib was decided by the treating oncologist, based on the patient’s performance status and co-morbidities. No concurrent chemotherapy was given with pazopanib, palliative radiotherapy (for pain control or haemostasis)/palliative surgery like amputation was allowed if indicated. Response assessment was defined as per Response Evaluation Criteria In Solid Tumours (RECIST) 1.1 criteria [13]. Progression was documented by clinical and/or radiological progression. The best response achieved at any time point throughout treatment with pazopanib was documented. Radiological responses were documented as complete response, partial response (PR), stable disease (SD) or progressive disease (PD).

Grade 3 and Grade 4 treatment-related adverse events (TRAEs) were assessed as per National Cancer Centre Common Terminology Criteria for Adverse Events, version 4.03 [14]. Pazopanib was stopped in case of recurrent grade 3/4 toxicities despite dose modifications.

Baseline demographic and clinical profiles were part of descriptive statistics. Categorical variables were compared using chi-square or Fisher’s exact test. Continuous variables were compared by student *t*-test or rank-sum test. A *p*-value of <0.05 (one-sided) was considered statistically significant. Progression-free survival (PFS) was defined from the date of the start of pazopanib to the date of first documented progression (clinical and/or radiological). Overall survival (OS) was defined from the date of the start of the study drug to the date of death. Data were censored on 21 August 2020. PFS and OS were analysed using Kaplan–Meier survival curve estimates. The difference between groups was analysed using the log-rank test. All statistical computations were performed using STATA software (ver12 Stata Corp, College Station, TX, USA).

## Results

A total of 38 patients meeting the inclusion criteria between January 2013 and December 2019 were screened. Five patients with incomplete records were excluded. Thirty-three patients were included in the final analysis. Nine patients (27%) were initiated at 400 mg and 14 patients (43%) at 600 mg once daily and escalated if they tolerated well.

### Patient characteristics

The baseline characteristics of patients are elaborated in Table 1. The median age of patients for the entire cohort was 47 years (range, 18–85). Comorbidities were seen in 36% and 27% of the patients had upfront metastatic disease. Twenty-seven (82%) patients had received chemotherapy previously (one or more than one line, range 1–3). The majority (82%) of the patients had undergone prior surgery and 48% of patients received prior radiation therapy to the local site (either adjuvant or palliative).

### Outcomes

Nine patients (27%) achieved an objective response (all PR) and fourteen patients (42%) had SD as the best response documented (Table 2).

**Figure 1.**
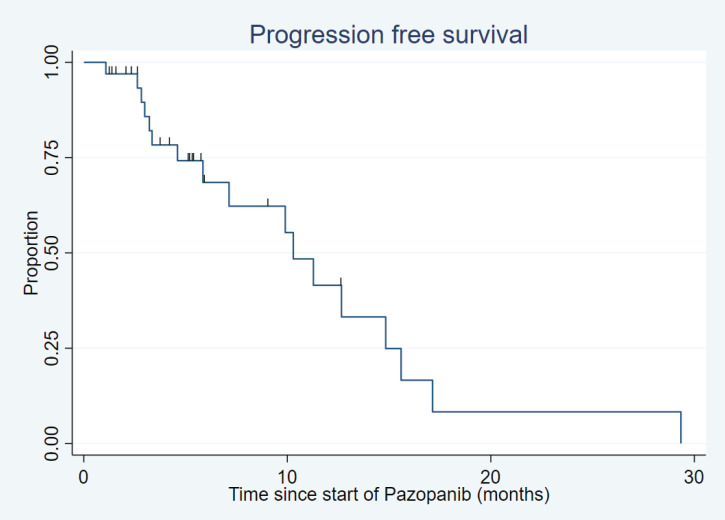
Progression-free survival.

**Figure 2.**
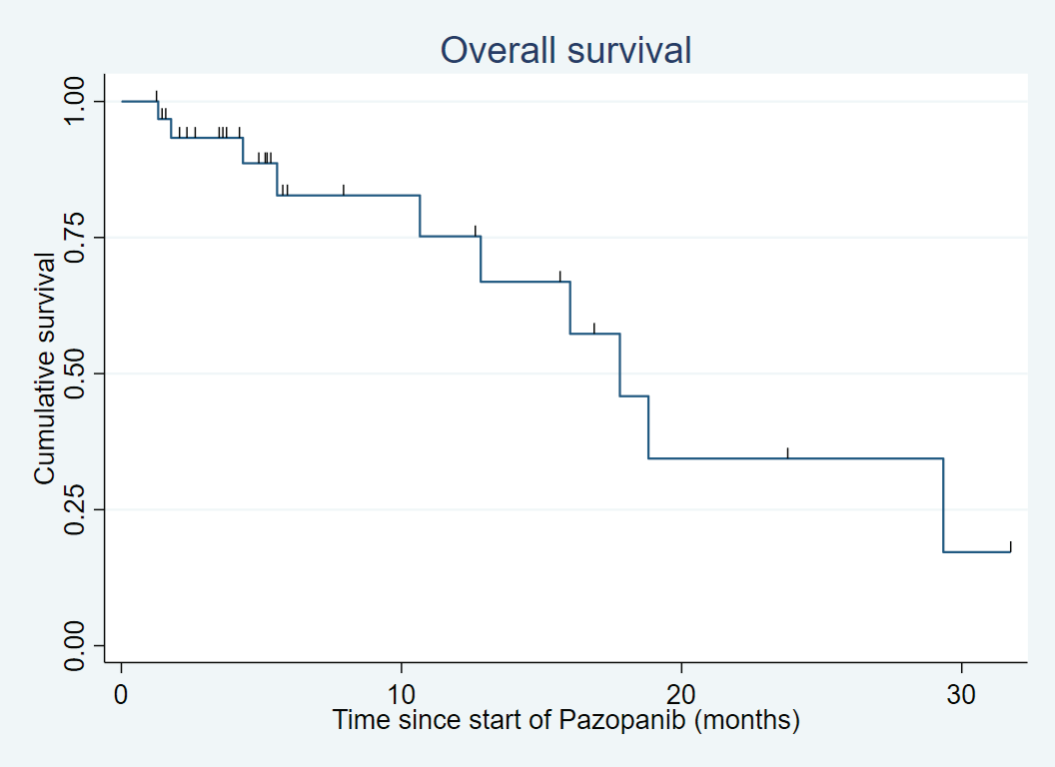
Overall survival.

After a median follow-up of 18.8 months (range: 1.9–150.4 months), the median PFS was 10.3 months (95% confidence interval (CI): 5.9–14.8) (Figure 1) and the median OS was 17.8 months (95% CI: 10.7–29.3) (Figure 2).

### Prognostic factors

On univariate analysis (Supplementary Table 1), only serum albumin (≤3.5) predicted for worse OS (Hazard Ratio (HR) – 2.39; 95% CI: 1.1–7.2, *p* = 0.005). No other factors (gender, primary site, Eastern Cooperative Oncology Group Performance Status (ECOG PS), haemoglobin, primary site of tumour, number of previous lines of treatment received or the starting dose of Pazopanib) were predictive for PFS or OS. Starting dose of Pazopanib didn’t predict for response rate.

### Subtype specific response

Of the 33 patients, we had seven cases of UPS, four cases each of MFS, epithelioid sarcoma and malignant peripheral nerve sheath tumour (MPNST), three cases each of haemangiopericytoma and spindle cell sarcoma, two cases of haemangioendothelioma and one case each of clear cell sarcoma, retroperitoneal sarcoma, angiosarcoma and pleomorphic rhabdomyosarcoma (RMS)-adult type, each (Table 1).

We have summarised subtype-specific response, PFS and OS for each case in Table 3.

### Toxicities

Grade 3 or 4 TRAE was observed in nine patients (27%). Mucositis, hand-foot syndrome and deep vein thrombosis were seen in two patients each, respectively. Other adverse events noted were gastritis, and diarrhoea in a patient (3%) each. Ten patients tolerated pazopanib well and were escalated to the full dose of 800 mg of pazopanib from the initial 400 mg dose. Four patients (12%) required dose modification for toxicities and 21% needed permanent discontinuation. There was no treatment-related mortality.

## Discussion

There is limited data available on the management of rare subtypes of STS. The role of chemotherapy remains doubtful if any [3]. There remains an unmet need for the management of the advanced, metastatic disease. Single-agent anthracycline-based chemotherapy is the first-line standard of care for unresectable/metastatic disease [7]. The management of rare STS subtypes has advanced from conventional chemotherapy to oral Tyrosine Kinase Inhibitors (TKIs) targeting angiogenesis. Most data, however, come from retrospective observational studies [3, 6, 9, 10, 11, 15], largely attributable to their rarity. Nevertheless, anti-vascular endothelial growth factor (anti-VEGF) TKIs like pazopanib has shown some activity in many of the rare STS subtypes [3, 11, 16–18].

At our centre, we use pazopanib for locally advanced unresectable or metastatic STS after progression on adriamycin-based first-line chemotherapy or in an upfront setting for patients unfit for chemotherapy.

In our cohort of 33 patients on pazopanib, we reported a longer median PFS of 10.2 months compared to the pazopanib for metastatic soft-tissue sarcoma (PALETTE) trial [19] and other reported studies on the use of pazopanib summarised in Table 4. This observed difference might be attributed to the indolent behaviour of these subtypes and/or their higher sensitivity to pazopanib. Most rare subtypes were excluded in the PALETTE trial, where pazopanib might have been more active than conventional chemotherapy. In addition to that, ethnicity might also play a role in observed differences in PFS.

Epithelioid sarcoma is refractory to conventional chemotherapy [15] with a reported median survival of 52 weeks with palliative chemotherapy[7]. Tazemetostat is a novel, small molecular inhibitor of enhancer of zeste homologue 2 recently approved by the Food and Drug Administration for treatment of advanced, metastatic epithelioid sarcoma, based on a phase II trial that included 62 patients and showed an objective response rate (ORR) of 15% with approximately two-third responses lasting more than 6 months [20]. Three out of four patients with epithelioid sarcoma in our study were alive on the data cut-off date (Table 3).

In clear cell sarcoma, characterised by t(12;22) (q13;q12) [21], anthracycline-based chemotherapy has limited activity with a median PFS of 11 weeks [22]. Studies suggest activity of kinase inhibitors like Crizotinib (CREATE trial) [23], Cabozantinib targeting Mesenchymal Epithelial Transition [24] and Pazopanib targeting VEGF pathways [11]. Our study included one patient with clear cell sarcoma who achieved SD and has not progressed until the data cut-off date (Table 3).

ASPS is characterised by the diagnostic unbalanced t(x;17)(p11,q25) translocation [25], wherein phase II studies have reported median PFS of 6 months on Pazopanib [16]. Immunotherapy trials involving Pembrolizumab have also has shown activity in advanced ASPS with 3 months reported PFS of 75% [26]. In our study, we had two patients of ASPS (Table 3), with results comparable to other reported studies.

There is doubtful efficacy of chemotherapy in haemangioendothelioma [27], retrospective observational studies have demonstrated the efficacy of anti-VEGF TKIs with ORRs ranging from 13% to 29%, with one study reporting median OS of 26 months in ten patients treated with pazopanib [18]. Of the two patients in our study, one had SD, while the other progressed (Table 3) on pazopanib.

In haemangiopericytoma (also known as solitary fibrous tumour), one report demonstrated a median OS of 14 months with palliative chemotherapy [28]. There is limited phase II data demonstrating the activity of anti-angiogenic agents like bevacizumab, sorafenib and sunitinib [29]. Martin-Broto *et al* [17] in their single-arm phase II prospective trial (*n* = 31) reported a median PFS of 9.8 months and mOS of 49.8 months on second-line Pazopanib. Of the three patients with haemangiopericytoma in our study (Table 3), two had SD and one progressed.

MFS and spindle cell sarcomas are unclassifiable subtypes of STS. MFS has a propensity for local recurrence with only case reports on palliative chemotherapy in metastatic disease [30]. We had seven such patients in our study (Table 3), out of seven patients, two had SD, two achieved PR and the rest had PD, showing proof of activity of Pazopanib in these subtypes.

UPS shows no definable line of differentiation and is a diagnosis of exclusion [5] and usually follows an aggressive clinical course with a poor prognosis. PALETTE trial included patients with UPS, however, subtype-specific PFS/OS was not reported [19]. The SARC28 trial of single-agent pembrolizumab treated ten patients of UPS among other subtypes and reported PR in four of ten patients [3]. Our study had seven patients of UPS (Table 3), two of whom achieved PR and four had SD.

We observed an ORR of 27%, all of which were PRs. Our results are comparable with other published reports on pazopanib (Table 5) which is better than chemotherapy for most subtypes as discussed above.

We observed a median OS of 17.8 months in our study cohort. Most published studies have either not reported median OS or had a short follow-up, where the median OS was not reached (Table 4). As discussed above, for most individual subtypes, the median OS observed in our study with pazopanib is better than observed with palliative chemotherapy. This encouraging results of the current study suggest that these histologies may be relatively more sensitive to pazopanib.

We started our patients on 400 mg pazopanib and escalated the dose based on tolerance. The rationale of this approach was based on our own country’s previous published experience with pazopanib in STS [31, 32] all of which reported poor tolerance of Indian patients to a starting dose of 800 mg pazopanib. Another study from India used pazopanib at a dose of 600 mg in combination with oral cyclophosphamide (50 mg once daily D1-21 q28 days) in patients with relapsed platinum-refractory carcinoma ovary reported dose reductions in 70% of patients as a result of toxicity [33]. Despite a starting dose of 400 mg, a significant number of patients in our study experienced grade 3 or higher toxicities with pazopanib, compared to the PALETTE trial [19], where maximum patients tolerated 800 mg dose. Our study findings, however, are similar to previous studies reported from India [31, 32]^’^ and Japanese Musculoskeletal Oncology Group study, where the average dose intensity of pazopanib was 609 mg and adverse events occurred in (81.4%) of the total cohort [34].^.^ Some factors responsible for higher toxicity in our patients may be attributed to lower body surface area and/or ethnic or geographic differences predicting poor tolerance in Asian patients.

Hypoalbuminaemia was the only prognostic factor for poor OS (Supplementary Table 1), which is in line with our previous experience in metastatic STSs and Ewing sarcoma [12, 35, 36].

 Our study is limited by its retrospective nature with a small sample size consisting of heterogeneous histopathological subtypes of uncommon STS. As STS of diverse histology were included, such a small sample size is inadequate to discern the selective activity of pazopanib for a particular subtype. Moreover, the molecular diagnostic tests were not carried out in all patients due to limitations in logistics, raising the possibility of misdiagnosis in a few cases.

The strength of our study lies in the compilation of a rather rare subgroup of STS and providing a proof of concept study of the activity of pazopanib in these rare, chemo-resistant STS subtypes where chemotherapy is futile. However, prospective data are required to confirm our observations.

## Conclusions

To conclude, the positive result of our study suggests that the rare subtypes of STS, which are usually chemo-resistant, may be relatively more sensitive to pazopanib. Pazopanib should be prospectively explored as a standard of care in this cohort.

## Conflicts of interest

There are no conflicts of interest.

## Funding statement

No funding was received for this research work.

## Authors’ contributions

All authors have participated in study design, data collection, data analysis and writing of the manuscript. All authors have given final approval of the version to be published.

## Institutional review

All authors have read and agreed to its content and the research work reported in the manuscript has been performed with the approval of the All India Institute of Medical Sciences, New Delhi, India ethics committee. Institute ethic committees’ reference no. IEC-115/01.02.2019 and EC/WV/TMC/15/21.

## Figures and Tables

**Supplementary Table 1.** Univariate analysis for prognostic factors.

Variable	Group	PFS	OS	Objective response (PR)
Gender	M F	HR – 0.45; 95% CI: 0.13–1.50, *p* = 0.19	HR – 1.5; 95% CI: 0.27–8.48, *p* = 0.49	-
ECOG PS	0–1 2–4	HR – 2.21; 95% CI: 0.76–6.40, *p* = 0.12	HR – 3.2; 95% CI: 0.87–12.48, *p* = 0.70	-
Primary site	Extremity Non-extremity	HR – 1.54; 95% CI: 0.35–6.68, *p* = 0.33	HR – 2.5; 95% CI: 0.37–16.84, *p* = 0.60	-
S.Albumin	<3.5 g/dL ≥3.5 g/dL	HR – 6.0; 95% CI: 0.81–43.9, *p* = 0.07	**HR – 2.3; 95%** **CI: 1.12–7.82, *p* = 0.005**	-
Haemoglobin	<10g/dL =≥10 g/dL	HR – 0.43; 95% CI: 0.10–1.75, *p* = 0.25	HR – 0.18; 95% CI: 0.01–2.10, *p* = 0.15	-
Pazopanib starting dose	400 mg/600 mg 800 mg	HR – 1.2; 95% CI: 0.33–4.32, *p* = 0.90	HR – 1.83; 95% CI 0.27–12.1, *p* = 0.33	HR – 7.0; 95% CI: 0.86–56.8, *p* = 0.069
Previous lines of therapy	<2 ≥2	HR – 0.88; 95% CI: 0.30–2.60, *p* = 0.82	HR – 1.52; 95% CI: 0.16–14.13, *p* = 0.7	-

M, Male; F, Female; ECOG PS, Eastern Cooperative Oncology Group Performance Status; PFS, Progression-free survival; OS, Overall survival; PR, Partial response; HR, Hazard ratio; CI, Confidence interval

**Table 1.** Patient characteristics (*N* = 33).

Characteristics	Patients receiving pazopanib (*N* = 33) n (%)
**Median age (range)**	47 (18–85)
**Sex** Male Female	18 (55) 15 (45)
**ECOG PS** 0 1 2	1 (3) 25 (76) 7 (21)
**Comorbidities** Yes No	12 (36) 21 (64)
**Site of disease** Extremities Axial Retroperitoneum Others^a^	20 (61) 9 (27) 2 (6) 2 (6)
**Baseline stage** Localised Metastatic	24 (72) 9 (27)
**Previous surgery** Yes No	27 (82) 6 (18)
**Previous radiation therapy** Yes No	16 (48) 17 (51)
**Previous lines of therapy** 0 1 2 3	11 (33) 19 (57) 2 (6) 1 (3)
**﻿Histopathology variant** ASPS Haemangiopericytoma Haemangioendothelioma MFS Spindle cell sarcoma MPNST Epithelioid sarcoma Clear cell sarcoma UPS Retroperitoneal sarcoma Angiosarcoma Pleomorphic RMS (adult type)	2 3 2 4 3 4 4 1 7 1 1 1

a Liver, breast

ECOG, Eastern Cooperative Oncology Group; PS, Performance status; MPNST, Malignant peripheral nerve sheath tumour; UPS, Undifferentiated pleomorphic sarcoma; RMS, Rhabdomyosarcoma

**Table 2.** Response and toxicity.

Characteristics	Pazopanib (*N* = 24) *N* (%)
Pazopanib dose (starting dose) 400 mg 600 mg 800 mg	9 (27) 14 (43) 10 (30)
Best response PR SD Progression	9 (27) 14 (43) 7 (21)
Grade III/IV toxicities	9 (27)
Dose modification needed	4 (17)

**Table 3.** Best response, PFS, OS of individual subtypes on pazopanib.

Subtype (Total *n* = 33)	Best response	PFS (months) on data cut-off date	Status/OS (months) on data cut-off date
**ASPS (*n* = 2)** Patient 1 Patient 2	PR SD	7.8 15	Alive (24) Alive (17)
**Haemangiopericytoma (*n* = 3)** Patient 1 Patient 2 Patient 3	SD SD PD	5.9 12.8 3.2	Alive (7.9) Dead (16.2) Alive (3.4)
**Haemangioendothelioma (*n* = 2)** Patient 1 Patient 2	PD SD	1.8 29.3	Dead (3.0) Dead (29.3)
**Myxofibrosarcoma (*n* = 4 )** Patient 1 Patient 2 Patient 3 Patient 4	PD PD SD SD	2.0 2.6 2.8 10.2	Alive (2.0) Dead (2.6) Alive (4.9) Dead (10.6)
**Spindle cell sarcoma (*n* = 3 )** Patient 1 Patient 2 Patient 3	SD PR PR	5.7 5.0 3.6	LFU (5.7) Alive (5.0) Alive (3.7)
**MPNST (*n* = 4)** Patient 1 Patient 2 Patient 3 Patient 4	PD SD PD NA	4.6 9.9 1.0 1.1	Alive (31.6) Dead (12.8) LFU (17.7) Alive (1.1)
**Epithelioid sarcoma (*n* = 4)** Patient 1 Patient 2 Patient 3 Patient 4	PR PR PR SD	12.5 2.2 3.4 5.1	LFU (12.6) Alive (2.2) Alive (3.6) Alive (5.1)
Clear cell sarcoma (*n* = 1)	SD	15.5	Alive (15.5)
**UPS (*n* = 7)** Patient 1 Patient 2 Patient 3 Patient 4 Patient 5 Patient 6 Patient 7	SD PR PR NK SD SD SD	11.2 5.2 4.1 1.3 17.3 8.9 5.3	Dead (18.8) Alive (5.2) Alive (4.1) Dead (1.3) Dead (17.5) LFU (8.9) Alive (5.3)
Retroperitoneal sarcoma (*n* = 1)	PD	2.6	Dead ( 4.3 )
Angiosarcoma (*n* = 1)	PR	5.8	Alive (5.8)
Pleomorphic RMS (*n* = 1)	NA	1.5	Alive (1.5)

PFS, Progression free survival; OS, Overall survival; ASPS, Alveolar soft part sarcoma; MPNST, Malignant peripheral nerve sheath tumour; UPS, Undifferentiated pleomorphic sarcoma; PR, Partial response; SD, Stable disease; PD, Progressive disease; NK, Not Known; NA, Not assessed; LFU, Lost to follow-up

**Table 4.** Studies reporting use of Pazopanib in uncommon/unclassified STS subtypes.

Author	No. of patients (n)	HPE subtype	ORR	PFS (months)	mOS (months)
Frezza *et al* [10]	18	Epithelioid sarcoma	0%	3	NR
Touati *et al* [8]	11	Epithelioid sarcoma	11.1%	3.8	10.8
Stacchiotti *et al* [37]	30	ASPS	24.2%	13.6	NR
Kim *et al* [16]	6	ASPS	16.7%	5.5	NR
Stacchiotti *et al* [38]	26	Extraskeletal myxoid chondrosarcoma	18%	2-year PFS – 40%	NR
Kollár *et al* [18]	52	Angiosarcoma, haemangioendothelioma Intimal sarcoma	20% 20% 100%	3 NR NR	9.9 NR NR
﻿Martin-Broto *et a*l [17]	31	Solitary fibrous tumour	58%	9.8	49.8
Our study	33	Haemangiopericytoma, pleomorphic RMS (adult type), MFS, spindle cell sarcoma, MPNST, ASPS, haemangioendothelioma, epethelioid sarcoma, clear cell sarcoma, UPS. Retroperitoneal sarcoma, angiosarcoma	27.2%	10.3	17.8

NR, Not reported; HPE, Histopathological examination; ORR, Objective response rate; PFS, Progression free survival; OS, Overall survival; ASPS, Alveolar soft part sarcoma; MPNST, Malignant peripheral nerve sheath tumour; UPS, Undifferentiated pleomorphic sarcoma; RMS, Rhabdomyosarcoma
